# Allelic imbalance at 1p36 may predict prognosis of chemoradiation therapy for bladder preservation in patients with invasive bladder cancer

**DOI:** 10.1038/sj.bjc.6602073

**Published:** 2004-08-03

**Authors:** H Matsumoto, H Matsuyama, K Fukunaga, S Yoshihiro, T Wada, K Naito

**Affiliations:** 1 Department of Urology, Yamaguchi University School of Medicine, 1-1-1, Minamikogushi, Ube, Yamaguchi, 755-8505, Japan

**Keywords:** bladder cancer, allelic imbalance, p73, p53, chemoradiation therapy, predictive marker

## Abstract

Invasive bladder cancers have been treated by irradiation combined with *cis-* platinum (CDDP) as a bladder preservative option. The aim of this study was to find a marker for predicting patient outcome as well as clinical response after chemoradiation therapy (CRT) by investigating allelic loss of apoptosis-related genes. A total of 67 transitional cell carcinomas of the bladder treated by CRT (median dose: 32.4 Gy of radiation and 232 mg of CDDP) were studied. We investigated allelic imbalances at 14 loci on chromosomes 17p13 and 1p36 including the *p53* and *p73* gene regions by fluorescent multiplex PCR based on DNA from paraffin-embedded tumour specimens and peripheral blood. The response to CRT was clinical response (CR) in 21 patients (31%), partial response (PR) in 31 (46%), and no change(NC) in 15 (22%). There was no statistical correlation between treatment response and clinical parameters, such as tumour grade, stage, radiation dose, or CDDP dose. The frequencies of allelic imbalance for TP53 and TP73 were 21 and 56%, respectively; neither was correlated with clinical treatment response and tumour stage or grade. There was no statistical correlation between treatment response and allelic imbalance at the other 12 loci. We found a significant correlation between cancer-specific survival and an imbalance of D1S243 (*P*=0.0482) or TP73 (*P*=0.0013) using a Log-rank test, although other loci including TP53 did not correlate with survival (*P*=0.4529 Multivariate analysis showed performance status (*P*=0.0047), recurrence (*P*=0.0017), and radiation doses (*P*=0.0468) were independent predictive factors for cancer-specific survival. However, an allelic imbalance of TP73 was the most remarkable independent predictive factor of poor patient survival (*P*=0.0002, risk ratio: 3382). Our results suggest that the allelic loss of the *p73* gene predicts a clinical outcome of locally advanced bladder cancer when treated by CRT.

Initial treatments for invasive bladder cancer can be divided into bladder-sparing therapy and radical cystectomy. Although radical cystectomy remains the standard treatment, a variety of adjuvant therapies have been attempted to induce tumour downstaging and to improve survival. During the last three decades, the most commonly used bladder-sparing treatment has been external beam radiation therapy (Shipley *et al*, 1987). Studies of systemic chemotherapy combined with radiation prior to surgery have shown that it can allow bladder preservation by transurethral resection (TUR) or partial cystectomy in 52–70% of patients with invasive bladder cancer (Tester *et al*, 1993; Tester *et al*, 1996; Kachnic *et al*, 1997; Shipley *et al*, 1997; Shipley *et al*, 1998). We have treated locally advanced bladder cancer by a modified version of Shipley's regimen using radiation combined with *cis-*platinum (CDDP) (Shipley *et al*, 1987). Although many tumours show a good response to this treatment, several are resistant. Since this therapy can be unnecessarily harmful for patients whose tumours do not respond, if we could predict the response of a bladder cancer to this treatment, it would help choose appropriate candidates for this therapy regime.

Dysfunction of the *p53* tumour suppressor gene located at 17p13.1 is the most frequent alteration in human malignancy and frequent loss of heterozygosity (LOH) on 17p13.3, distal to p53, has been reported in breast, colon, ovary, and lung cancers (Tsuchiya *et al*, 2000). Mutant P53 protein overexpression is frequently observed in bladder cancers that show more malignant behaviour. Loss of *p53* function is one of the most important roles in resistance to chemotherapy or radiation-induced apoptosis. The incidence of LOH of chromosome 17p or mutation of *p53* is 60% or more in advanced bladder cancer (Sidransky *et al*, 1991). However, more than a few patients with bladder cancer harbour *p53* alterations and do respond to CDDP. This finding suggests that there are some pathways other than *p53* activation that mediate CDDP-induced cell death, and that damage to these pathways leads to clinical crisis.

The *p73* gene that has been mapped to chromosome 1p36.3, which has a high homology to the *p53* gene, is also a key factor in the apoptotic pathway. It has been reported that *p73* is a component of a mismatch repair-dependent pathway, and that *p73* levels are increased after DNA damage (Agami *et al*, 1999; Gong *et al*, 1999; Yuan *et al*, 1999). Many studies have suggested that there may be several tumour suppressor genes on 1p36, such as in neuroblastoma and hepatocellular carcinoma (Ichimiya *et al*, 1999; Fang *et al*, 2000).

This evidence prompted us to investigate specific loci encompassing *p53* and *p73* genes by microsatellite analysis using the fluorescent multiplex PCR technique, and to investigate genetic markers that could help predict the clinical outcome of chemoradiotherapy in bladder cancer.

## MATERIALS AND METHODS

### Patients

A cohort of 67 patients, who underwent preoperative chemo-radiotherapy (CRT) for locally invasive (T2-4N0M0) or high-risk superficial (pT1G3) bladder cancer between November 1994 and August 2000, was studied (Table 1

**Table 1:** Patient characteristics

**Characteristics**	**No.**	**%**
All patients	67	
*Sex*		
Male	51	76
Female	16	24
*Age (years)*		
Median	68	
Range	45–89	
*Histopathology*		
Transitional cell carcinoma	67	100
*Pathological grade*	15	22
G2	52	78
G3		
*Clinical stage*		
T1&G3	12	18
T2	22	33
T3	28	42
T4	5	7
*Lymph node metastasis*		
N(+)	0	0
N(−)	67	100
Median follow-up period (months)	32.6	

). Before treatment, we performed cold cup biopsies on tumours and selected-site bladder mucosa that were taken from trigone, both lateral walls, retrotrigone, dome, anterior wall, bladder neck, and prostatic urethra in men. Systemic work-up for tumour staging on CT scanning, intravenous pyelography, and bone scintigram were performed for all patients. Tumours were graded histopathologically according to the WHO classification and were staged by the TNM staging system of the UICC (1992). All patients received preoperative chemotherapy with *cis*-diamino-dichloro-platinum (CDDP) intravenously plus radiotherapy. The regimen (based on Shipley's method) was CDDP (70 mg m^−2^) on day 1, with radiation administered by Liniac to the true pelvis at 1.8 grays (Gy) per fraction from day 2 to day 6 in the first week, and 5 consecutive days per week thereafter. The therapy was carried out one to three times every 14 days. The mean total dose was 40.5 Gy, ranging from 30 to 61.2. At the completion of one cycle of CRT, patients were treated by CDDP of 70 mg m^−2^ and irradiation of 16.2 Gy to the basic target. Although we tried to perform three cycles of CRT when possible, in those patients who received one or two cycles of the therapy and who had persistent side effects such as nausea, vomiting, diarrhoea, and pancytopenia for 2 weeks or who refused to continue with CRT, the treatment was halted. At 4 weeks after the completion of CRT, patients were assessed for response by selected-site mucosal cold cup biopsy or TUR and CT scanning. The clinical response to CRT was classified as follows: CR (clinical response), pathologically no tumour residual in the bladder (pCR) and no evidence of nodal or visceral metastasis; PR (partial response), downsizing of the tumour with a decrease of over 50% compared to the initial tumour, with no evidence of distant or nodal spread on CT scanning. Cases that were pathologically not pCR were classified as PR even if the tumour showed complete disappearance radiographically. NC (no change): persistent invasive disease that was not downsized by CRT, with or without evidence of nodal or distant spread. Patients whose tumour showed NC or whose residual tumour cells remained in the muscle layer were referred for radical or partial cystectomy with lymph node dissection, while patients with responding tumours (CR or PR) underwent complete resection of the cancer by TUR. Six patients who had just residual carcinoma *in situ* (CIS) were subsequently treated by intravesical instillation of Mitomycin C (MMC) or BCG. Treatment-related toxicities were evaluated by WHO criteria. Cystoscopic examination followed by washing cytology was carried out every 3 months from the third to fifth year and every 6 months thereafter. Complementary examinations, including chest X-rays and/or CT scans, were carried out every 6 months.

### Specimens and DNA extraction

Specimens of tumours collected at the time of initial diagnosis were studied for the source of tumour DNA. Formalin-fixed paraffin-embedded tissues were cut into 5-*μ*m sections, and the first and last sections were used for histopathological analysis with HE stain. Specimens containing at least more than 75% tumour cells were used for analysis. Specimens from three-step sections were used for DNA extraction using DEXPAT (Takara, Tokyo, Japan) according to the manufacturer's instructions.

### Blood collection

Blood samples from each patient were collected as a normal control. Blood was treated with EDTA, and lymphocyte DNA was isolated using a GenTLE extraction kit (Takara, Tokyo, Japan).

### PCR amplification

Totally, 14 microsatellite markers containing loci on chromosome 17p13 and 1p36, with two- or three-base pair repeat motifs were investigated. The primer sequences for these markers were obtained from the Genome Database (http://gdbwww.gdb.org). The location of each marker is shown in Table 2

**Table 2:** Prevalence of allelic imbalance, and observed heterozygosities at different loci in lp and 17p

		**Allelic imbalance**		
**Marker**	**Locus**	**No/Informative case (%)**		**Observed heterozygosities**
1 D1S171	Ip36.3	19/55	(34.5)	0.78
2 TP73	lp36.2–36.3	32/57	(56.1)	0.86
3 D1S243	lp36.2–36.3	18/57	(32.1)	0.86
4 D1S244	lp36.2–36.3	19/57	(32.8)	0.74
5 RIZ	Ip36.2	22/56	(39.3)	0.68
6 D1S507	Ip36.1–36.2	20/57	(35.1)	0.83
7 D1S247	Ip35–36.1	24/57	(42.1)	0.81
8 ABR	17pl3.3	28/57	(49.1)	0.96
9 D17S1574	17pl3.3	15/56	(26.8)	0.68
10 YNZ22	17pl3.3	19/54	(35.2)	0.63
11 D17S1566	17pl3.2–13.3	12/57	(21.1)	0.72
12 ENO3	17pl3.2	18/55	(32.7)	0.89
13 TP53	17pl3.1	12/57	(21.1)	0.72
14 D17S520	17pl2–13.1	15/58	(25.9)	0.74

. Polymerase chain reaction (PCR) was carried out in a reaction volume of 10 ml containing 0.2 mM of each primer (one of the primers was labelled with FAM, HEX, or TET), 2 mM MgCl_2_, 0.2 mM dNTP, 1 U of *Taq* polymerase, and approximately 5 ng of genomic DNA. Amplification was carried out by 40 PCR cycles of 94°C for 45 s, 55°C for 60 s, and 72°C for 45 s.

### Multiplex PCR

The multiplex PCR method was the same as described above, with the exception that 2–5 primer pairs were added in the same PCR tube. Primers were selected on the basis that the resultant PCR products could be distinguished by colour and size without overlapping.

### Polyacrylamide gel electrophoresis

The PCR products were subjected to electrophoresis on 4% polyacrylamide denaturing gel in TBE buffer and analysed using an automated fluorescent DNA sequencer (model 377, Applied Biosystems, CA, USA). In total, 1 *μ*l of each PCR product was resuspended using 3.5 *μ*l of loading solution (2.5 *μ*l formamide, 0.5 *μ*l of 50 mM EDTA with 50 mg ml^−1^ Blue Dextran, and 0.5 *μ*l of Gene Scan Size Standard: GS500). This mixture was denatured at 95°C for 5 min and a 1.5 *μ*l aliquot of the mixture was used for analysis. Electrophoresis was performed for 2 h at 40 W and 52°C.

### Data analysis and calculation of allele ratios

To detect allelic imbalance, the peak area of the two alleles was calculated by Gene Scan Analysis Software for each tumour specimen and the corresponding lymphocyte DNA. Allelic imbalance was defined as a decrease of the signal of one allele by more than 50% in tumour DNA when compared with lymphocyte DNA.

### Statistical analysis

Correlations of frequency of allelic imbalance with tumour grade, stage, and response to CRT were assessed using *χ*^2^ or Fisher's exact tests. Survival after CRT was analysed using the Kaplan–Meier method with a Log-rank test as univariate analysis. Multivariate analysis was performed by using Cox's proportional hazard regression test in step-wise mode. Data were processed using JMP 4.0 statistical software, with *P*<0.05 indicating statistical significance.

### Ethical considerations

Our research was carried out in accordance with the principles set out in the Declaration of Helsinki 1964 and all subsequent revisions, informed consent was obtained, and the ethical review committee of Yamaguchi University School of Medicine approved the study.

## RESULTS

### Treatment response and prognosis

A total of 67 patients (median age: 68 years; range: 45–89) received CRT with a median follow-up period of 32.6 months (range: 2.9–84.2) and were eligible for evaluation (Table 1). The average total dose of CDDP and radiation was 230 mg (median 220 mg, range: 75–375) and 40.5 Gy (median 32.4, range: 30–61.2), respectively. Treatment-related toxicity such as anemia, leucocytopenia, thrombocytepenia, nausea, vomiting, and diarrhoea were mild and tolerable. Severe toxicity over grade 3 was not observed. The response to CRT was CR in 21 patients (31%), PR in 31 (46%), and NC in 15 (22%). Recurrence was noted in 26 patients (39%), including 11 with progressive disease. Of the patients who achieved pCR, nine (42.8%) had recurrence. Three patients had superficial local recurrence, and six had invasive local recurrence or distant metastases. A total of 15 patients suffered cancer-related death.

No statistical relation was found when treatment response (CR or non-CR) was compared with tumour stage, grade CDDP doses, or radiation doses (Table 4).

### Deletion mapping of chromosome 1 and chromosome 17

**Figure 1:**
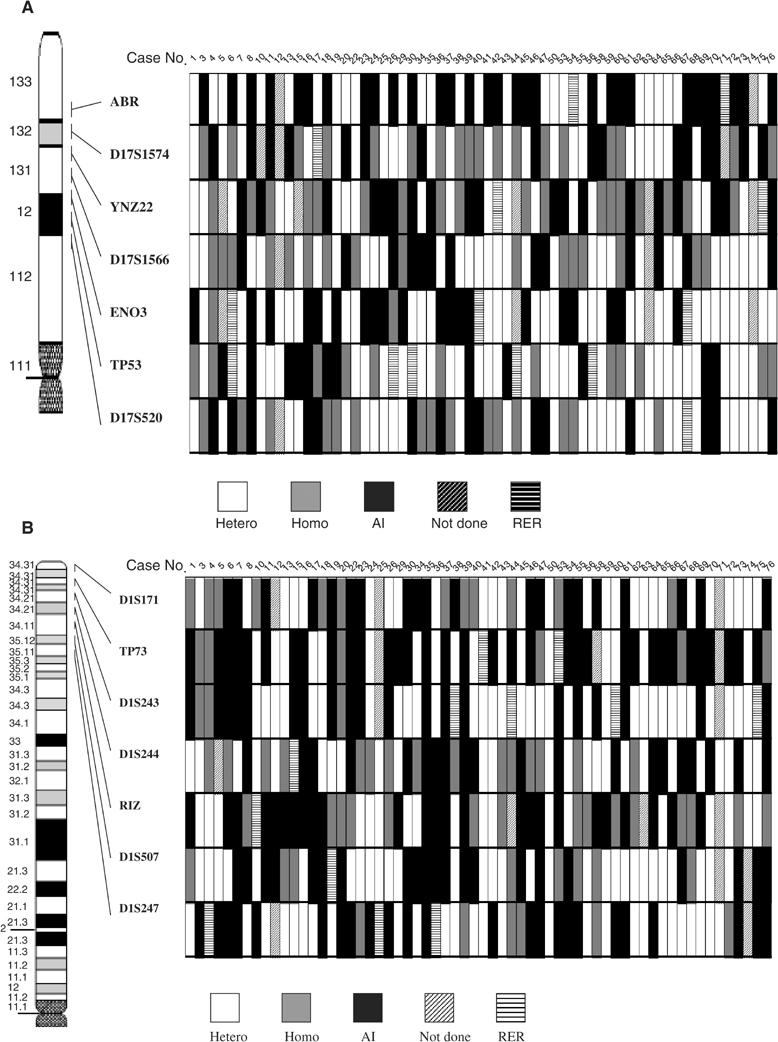
Deletion mapping on chromosomes 17p13 (**A**) and 1p36 (**B**) in bladder cancer. Hetero, heterozygote; Homo, homozygote; AI, allelic imbalance; RER, replication error.

Table 2 shows the prevalence of allelic imbalance and the fraction of heterozygosity, which ranged between 0.68 and 0.96. Figure 1

summarises the results of allelic imbalance of the 14 microsatellite markers. Not done was defined either as when DNA amplification failed for technical reasons, or when the specific chromosomal allele did not show heterozygosity with the control lymphocyte DNA. Of the 67 tumours, allelic imbalance was observed from 21.1 to 56.1% in 14 markers. A significantly higher frequency of allelic imbalance was observed in the *p73* locus than those at the *p53* locus (56.1 *vs* 21.1%, *P*=0.003).

### Relation of allelic imbalance to clinicopathological parameters and treatment response

Table 3

**Table 3:** Correlation of grade and stage to frequency of allelic imbalance

		**Histological grade**			**Clinical stage**		
		**G2/G3(%)**	***P***-**value**		**Tl*1*T2/T3 & T4 (%)**	***P***-**value**	
1	D1S 171	9.1/25.5	0.4845	NS	3.6/14.6/16.4	0.6891	NS
2	TP73	13.0/44.4	0.5014	NS	12.3/15.8/28.1	0.1755	NS
3	D1S243	5.4/26.8	1.0000	NS	5.4/10.7/16.1	0.8545	NS
4	D1S244	5.4/26.8	0.7320	NS	5.4/8.9/17.9	0.6173	NS
5	RIZ	5.4/33.9	0.3287	NS	5.4/17.9/16.1	0.6118	NS
6	D1S507	7.0/28.1	1.0000	NS	0/17.5/17.5	0.0305	
7	D1S247	8.8/33.3	1.0000	NS	7.0I10.5/24.6	0.2440	NS
8	ABR	12.3/36.8	0.6982	NS	8.8/14.0/26.3	0.4749	NS
9	D17S1574	7.1/19.6	0.7145	NS	3.6/10.7/12.5	0.8435	NS
10	YNZ22	7.4/25.9	1.0000	NS	3.7/11.1/18.5	0.5769	NS
11	D17S1566	5.3/15.8	1.0000	NS	1.8/8.8/10.5	0.5492	NS
12	ENO3	5.5/29.1	0.5114	NS	5.5/10.9/18.2	0.8332	NS
13	TP53	3.6/17.9	1.0000	NS	3.5/8.8/8.8	0.9198	NS
14	D17S520	7.0/17.5	0.2882	NS	3.5/8.6/13.8	0.7008	NS

NS=not significant.

shows the relationship of allelic imbalance to tumour stage and grade. Except for the D1S507 locus, no statistically significant association was found between allelic imbalance and tumour stage or grade at any locus. At the D1S507 locus, allelic imbalances were observed at a significantly high frequency in the progressive stage (*P*<0.0305).

As for the relationship between allelic imbalance and treatment response, significant differences of allelic imbalance were not observed between CR and non-CR patients at each locus (Table 4

**Table 4:** Correlation of treatment response to frequency of allelic imbalance and clinical parameters

		**Variable**	**CR**	**Non-CR**		***P*-value**	
Microsatellite markers	1	D1S171	7.3	27.3	(%)	0.1489	NS
	2	TP73	16.7	40.7		0.6953	NS
	3	D1S243	12.5	19.6		0.5895	NS
	4	D1S244	8.9	23.2		0.7633	NS
	5	RIZ	16.1	23.2		0.3748	NS
	6	D1S507	12.3	22.8		0.9919	NS
	7	D1S247	8.8	33.3		0.0878	NS
	8	ABR	12.3	36.8		0.1897	NS
	9	D17S1574	8.9	17.9		1.0000	NS
	10	YNZ22	9.3	24.1		0.7635	NS
	11	D17S1566	5.3	15.8		0.7338	NS
	12	ENO3	9.1	25.5		0.5542	NS
	13	TP53	8.9	12.5		0.5230	NS
	14	D17S520	6.9	19.0		0.7515	NS
Clinical parameters							
		G2 or G3	31.3	68.7	(%)	0.7601	NS
		TlorT2orT3&T4	31.3	68.7		0.3066	NS
		CDDP dose^a^	216	237	(mg)	0.2107	NS
		Radiation dose^a^	40.9	40.5	(Gv)	0.8665	NS

a Mean dose.

CR=clinical response; CDDP=*cis-*platinum; NS=not significant.

).

### Correlation between cause-specific survival and allelic imbalance

Table 5

**Table 5:** Univariate and multivariate analyses for predicting cause-specific survival in bladder cancer patients treated with chemoradiotherapy

	**(a) Univariate analysis^a^**				**(b) Multivariate analysis^b^**
	**Variable**	**Category**	***P*-value**		**Risk ratio (95% CI) *P*-value**
	D1S 171	Balance or imbalance	0.2588	NS	NS
Microsatellite markers	TP73		0.0013		3382(not calculated) 0.0002
	D1S243		0.0482		NS
	D1S244		0.8444	NS	NS
	RIZ		0.6178	NS	NS
	D1S507		0.2756	NS	NS
	D1S247		0.9640	NS	NS
	ABR		0.2051	NS	NS
	D17S1574		0.1859	NS	NS
	YNZ22		0.3886	NS	NS
	D17S1566		0.5149	NS	NS
	ENO3		0.7176	NS	NS
	TP53		0.4529	NS	NS
	D17S520		0.2632	NS	NS
					
Clinical parameters	Age	⩽68 years or >68 years	0.6652	NS	NS
	PS	0 or >0	0.0113		2.911(1.31–12.38)0.0047
	Tumour grade	G2 or G3	1.0000	NS	NS
	Tumour stage	Tl or T2 or T3 & T4	0.5989	NS	NS
	Recurrence	Yes or No	0.0027		3.467(1.50–14.90)0.0017
	Response	CR or non-CR	0.4916	NS	NS
	CDDP doses	⩽220 mg or >220 mg	0.3634	NS	NS
	Radiation doses	⩽32.4 Gy or >32.4 Gy	0.0486		1.655(1.007–2.935) 0.0468

a Used Log-rank test.

b Used the Cox proportional hazard test.

**Figure 2:**
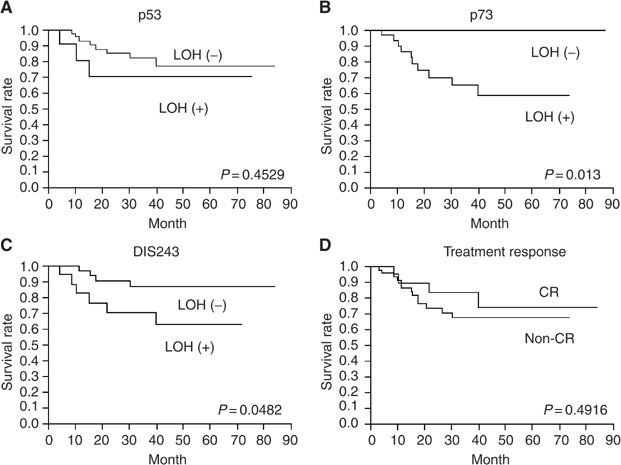
Kaplan–Meier survival curves stratified by TP53 (**A**), TP73 (**B**), D1S243 (**C**), and treatment response (**D**).

CDDP=*cis-*platinum; CR=clinical response; NS=not significant.

shows the correlation between cause-specific survival and candidate-predictive factors including allelic imbalance at each locus. Significant correlation was observed between allelic imbalances of TP73 (imbalance: death 10 *vs* alive 22; balance: death 0 *vs* alive 25, *P*=0.0013) or its neighbour locus, D1S243 (imbalance: death 6 *vs* alive 12; balance: death 4 *vs* alive 34, *P*=0.0482) and survival rate; whereas no association was observed at TP53 (imbalance: death 3 *vs* alive 9; balance: death 8 *vs* alive 37, *P*=0.4529). Clinical parameter, grade, stage treatment response; CR or non-CR (imbalance: death 4 *vs* alive 17; balance: death 13 *vs* alive 33, *P*=04916), and CDDP dose did not correlate with cause-specific survival, although significant correlation was observed between survival rate and recurrence or no recurrence (*P*=0.0027), performance status (0.0113), and radiation doses (*P*=0.0486), respectively. By multivariate analysis before treatment, an allelic imbalance of TP73 was the most useful independent predictive factor in predicting cause-specific survival in bladder cancer patients treated by CRT (Table 5, *P*=0.0002, Risk ratio: 3382). Figure 2

plots the Kaplan–Meier's survival curves stratified by treatment response, and allelic imbalance of TP73, D1S243, and TP53.

## DISCUSSION

We applied a newly developed fluorescent multiplex-PCR technique, which achieves superior resolution when compared with isotopic methods, to microsatellite detection. We found loss of heterozygosity (LOH) at many loci on chromosomes 1 and 17 in 67 bladder cancers. Previous studies have identified LOH at 3p, 4p, 4q, 6p, 8p, 9p, 9q, 10q, 11p, 13q, 14q, 17p, and 18q in advanced bladder cancers (Tsai *et al*, 1990; Reznikoff *et al*, 1996). Close association of LOH found at specific locations to prognosis as well as chemotherapy response was reported in other malignancies (Feugeas *et al*, 1996). Sengelov *et al* (2000) reportedly found no predictive markers including LOH at 1p, 8p, 10p, 13q, and 17p for response to chemotherapy in advanced bladder cancer. Their data is in contrast with our data showing the close association of allelic imbalance at *p73* with cause-specific survival in patients who were treated by CRT in bladder cancer, although the prevalence of allelic imbalance at *p73* did not correlate with treatment response. We consider that their series analysed patients with more advanced disease (metastatic or recurrent) and with resistant to various treatments before chemotherapy; thus, having a much worse prognosis than the group of patients in our study. Sengelov's series might have had more heterogeneous changes of genes than that of our series. In our study, patients were only locally advanced primary treated cases and not recurrent or metastatic cases. Therefore, we believe that their series would not have shown a significant correlation between allelic imbalances and response to chemotherapy or duration of survival.

LOH at 17p is common, 40–63%, in patients with advanced bladder cancer (Olumi *et al*, 1990; Tsai *et al*, 1990; Dalbagni *et al*, 1993; Reznikoff *et al*, 1996). Many tumours with LOH at the 17p locus have been shown to have mutations in the retained 17p allele (at the *p53* locus) affecting mutated P53 protein overexpression. *p53* is one of the most important genes in the tumour suppressor or regulator cell cycle and apoptosis. In addition, expression of *p53* is induced by several types of DNA damage caused by CDDP or irradiation. LOH at 17p (including the *p53* locus) have been shown to predict a poor response to chemotherapy in head and neck cancer. In contrast to these data, our results are in good agreement with another study showing a lack of relation between *p53* mutation and chemotherapy response (Sengelov *et al*, 2000).

Another region of interest is on 17p13.3, telomeric to the TP53 locus in several types of tumours, where the presence of new tumour suppressor genes is indicated in hepatocellular carcinoma, malignant astrocytoma, paediatric primitive neuroectodermal tumours, breast cancer, high grades of astrocytic tumours, and ovarian cancer (Biegel *et al*, 1992; Saxena *et al*, 1992; Nishida *et al*, 1993; Isomura *et al*, 1994; Phillips *et al*, 1996; Chattopadhyay *et al*, 1997; Haataja *et al*, 1997). In lung cancer, new tumour suppressor gene(s) may reside on three distinctly deleted regions on chromosome 17p13.3 distal to the *p53* gene, with possible roles in progression and differentiation of adenocarcinomas (Tsuchiya *et al*, 2000). Thus, we expected that new tumour suppressors or apoptosis-related genes may reside in 17p13 regions and investigated LOH of the TP53 locus and its distal regions. However, we did not find any correlation between the prevalence of LOH at each locus and tumour grade, stage, or response to CRT. In our study, the prevalence of LOH at the TP53 locus was not very frequent. These results suggest that LOH study at the *p53* gene and its neighbour locus is not important for predicting chemoradiosensitivity or survival in locally advanced bladder cancer. However, loss of function of *p53* by genetic or epigenetic changes is a most important factor in the malignant potential of bladder cancer, and further examination is needed to predict the clinical course treated by CRT.

The distal region of the short arm of human chromosome 1 (1p36) is thought to contain tumour suppressor genes, such as *p73*and RIZ, in a variety of cancers. The *p73* gene, a member of *p53* gene family, is also an important gene reflecting apoptosis or tumorigenesis in bladder cancer. The *p73* gene has been mapped at chromosome 1p36.3, a region frequently deleted in breast cancer, neuroblastoma, and other malignancies (Han *et al*, 1999; Ichimiya *et al*, 1999; Ahomadegbe *et al*, 2000). RIZ, retinoblastoma protein (Rb)-interacting zinc-finger gene (PRDM2), was isolated during functional screening for Rb-binding proteins (Buyse *et al*, 1995) and was mapped to 1p36.2; the RIZ locus is a target of frequent deletion in hepatocellular carcinoma (Fang *et al*, 2000). Several reports have suggested that *p73* is not likely to be a tumour suppressor gene, and that its overexpression by the activation of a silent allele may contribute to the progression of bladder cancer (Chi *et al*, 1999; Yokomizo *et al*, 1999). In contrast, Ono *et al* (2001) demonstrated that the absence of CDDP-induced apoptosis in p53-defective TCC cells, established CDDP-resistant sublines from the TCC cell line T24, may be attributable to a loss of *p73* induction that would be followed by insufficient caspase activation. Our results are partially consistent with their data, leading to the hypothesis that inactivation of *p73* may be the cause of acquired resistance to CDDP observed in TCC in clinical settings.

It is likely that *p73* overexpression may occur during an early to intermediate phase of bladder cancer. Cancer cells at that time acquire a more malignant behaviour, and they could lose one allele at many loci, including the *p73* gene, due to genomic instability at an advanced stage of bladder cancer. Indeed, overexpression of *p73* gene may be unnecessary for cancer cell survival in a late phase.

In conclusion, our results suggest that the allelic imbalance at *p73* may be a useful prognostic indicator in bladder cancer patients treated by CRT. This may enable appropriate candidates for CRT to be selected from among patients with locally advanced bladder cancer. Patients with an allelic imbalance at *p73* may be considered for immediate cystectomy, rather than for bladder preservation by CRT. Investigation of a larger number of patients with additional follow-up treatment by CRT is required to validate our preliminary results.
